# Stimulus waveform design for decreasing charge and increasing stimulation selectivity in retinal prostheses

**DOI:** 10.1049/htl.2019.0115

**Published:** 2020-06-23

**Authors:** Pragya Kosta, Kyle Loizos, Gianluca Lazzi

**Affiliations:** 1 Department of Electrical and Computer Engineering, University of Utah, Salt Lake City, UT 84112, USA; 2 Department of Electrical Engineering, University of Southern California, Los Angeles, CA 90089, USA; 3 Department of Ophthalmology, University of Southern California, Los Angeles, CA 90033, USA

**Keywords:** prosthetics, biological tissues, neurophysiology, cellular biophysics, eye, bioelectric potentials, diseases, tissue damage, proximal ganglion cells, degenerated retina, electrical stimulation, multiscale computational model, temporal spiking patterns, retinal prosthetic devices, ganglion cell activity, oscillatory behaviour, cone bipolar cells, photoreceptors, presynaptic input, photoreceptor layer, Retinitis pigmentosa, retinal degenerative diseases, retinal prostheses

## Abstract

Retinal degenerative diseases, such as retinitis pigmentosa, begin with damage to the photoreceptor layer of the retina. In the absence of presynaptic input from photoreceptors, networks of electrically coupled AII amacrine and cone bipolar cells have been observed to exhibit oscillatory behaviour and result in spontaneous firing of ganglion cells. This ganglion cell activity could interfere with external stimuli provided by retinal prosthetic devices and potentially degrade their performance. In this work, the authors computationally investigate stimulus waveform designs, which can improve the performance of retinal prostheses by suppressing undesired spontaneous firing of ganglion cells and generating precise temporal spiking patterns. They utilise a multi-scale computational model for electrical stimulation of degenerated retina based on the admittance method and NEURON simulation environments. They present a class of asymmetric biphasic pulses that can generate precise ganglion cell firing patterns with up to 55% lower current requirements compared to traditional symmetric biphasic pulses. This lower current results in activation of only proximal ganglion cells, provides more focused stimulation and lowers the risk of tissue damage.

## 1 Introduction

Millions of people have vision impairment due to diseases such as glaucoma, diabetic retinopathy, age-related macular degeneration, and retinitis pigmentosa (RP) [1]. In the last few decades, visual prosthetic systems have garnered significant interest, as they have shown the potential to restore useful vision by external stimulation of the retina or visual cortex [2–8]. Although cortical implant-based visual prosthetic systems hold the potential to treat multiple blindness conditions, these systems are currently under development [7, 8]. On the other hand, various retinal prosthetic systems, such as epiretinal, subretinal, and suprachoroidal retinal implants, have passed first-in-human clinical trials and currently being researched for further performance improvements [3, 4, 6].

Retinal prosthetic systems aim to provide treatment for retinal degenerative diseases, such as RP. In RP, although photoreceptors are substantially damaged, internal retinal neurons and their circuitry remain mostly intact [2]. External electrical stimulation of these surviving retinal neurons has proven capable of restoring partial vision [3]. Retinal prosthetic devices help patients perform daily activities; however, the restored vision is not comparable with the visual perception produced by a healthy retina [3]. The performance of retinal prostheses is hindered by various challenges, including limited implant electrode density, lack of stimulation selectivity of target neuronal population, and reduction in the sensitivity of neurons to electrical stimulation over time [9]. Another factor that could be responsible for the degraded performance of retinal prostheses is the spontaneous activity observed in the degenerated retina [10–12]. In the absence of presynaptic input from photoreceptors, electrically coupled AII amacrine cell and bipolar cell networks show oscillatory behaviour and lead to the spontaneous firing of ganglion cells [10].

Various model-based and experimental studies have been conducted to explore the possibilities of improving the electrical stimulation of the retina [9]. Multiple research groups have explored the stimulus waveform designs to enhance stimulation efficiency [13–18]. Adjustments in the design parameters of symmetric biphasic pulses, such as stimulation duration and inter-phase gap, have been shown to reduce the stimulation threshold [13]. Other waveform shapes, such as Gaussian and sinusoidal stimulus waveforms, have been shown to decrease peak current densities at the electrode–tissue interface, reducing electrode corrosion and tissue damage [14, 15]. Additionally, stimulation using asymmetric biphasic waveforms has been found to help in reducing stimulation thresholds [16]. Studies have suggested that rectangular stimuli might not be optimal for stimulation of retinal ganglion cells, with non-rectangular waveforms being shown to elicit stronger responses with higher charge-efficiency [17, 18].

Unconventional waveform designs have also been explored to improve selective stimulation of specific neuronal populations inside retinal cell networks [19–24]. Sinusoidal waveforms have been suggested to preferentially activate photoreceptors, bipolar cells, or ganglion cell populations [19, 20]. Furthermore, asymmetric biphasic waveforms have been found to provide control over direct versus indirect activation of retinal cells [21]. Other studies have suggested that the duration of stimulation can be utilised to selectively activate between retinal ganglion cells, bipolar cells, and amacrine cells [22], modulate the selectivity for ON versus OFF responses [23] and produce precise temporal patterns of ganglion cell firing [24].

These studies explore stimulus waveform designs towards improvement in the performance of retinal stimulation. However, spontaneous neural activity, which is an inherent characteristic of the degenerated retina, is not considered. Recently, Haselier *et al.* showed that electrical stimulation efficiency is strongly reduced in the degenerated retina when oscillations and rhythmic firing of cells are observed [25]. The authors also reported that usage of a pre-stimulation pulse sequence could enhance the stimulation efficiency. Nonetheless, this pre-stimulation sequence could only help in some cases and for a limited time [25].

In this work, we focus on suppressing the effect of spontaneous ganglion cell activity on the performance of electrical stimulation through stimulus waveform design. Specifically, leveraging our multi-scale computational framework [26, 27], we introduce here a new class of stimulus waveforms that can suppress the uncontrolled firing of ganglion cells due to spontaneous activity and can generate precise temporal spiking patterns. We tested four different asymmetric biphasic waveform shapes, each with a short cathodic phase for stimulation and a long anodic phase for charge recovery. Each waveform has a different rate of charge recovery during the anodic charge reversal phase. All the waveforms are charge-balanced to incorporate the basic requirement of tissue safety. We compared the performance of these waveforms against a symmetric biphasic pulse and analysed them in the following aspects: (i) ability to suppress the spontaneous firing of ganglion cells, (ii) stimulation selectivity, and (iii) tissue safety.

## 2 Methods

Stimulus waveforms are characterised using a simulation framework that models the electrical stimulation of degenerated retina and predicts the response of retinal cells. The simulation framework comprises a bulk tissue model and a network of retinal cells.

A 6.25 × 4.25 mm section of retina tissue is modelled using a heterogeneous multi-resolution tissue model. The tissue model consists of various retinal layers, and the thickness of each layer is assigned such that the model replicates an early stage degenerated retina tissue [28]. The model of an electrode array of 6 × 10 electrodes, each with a diameter of 200 μm, is positioned on the retina's inner surface (epiretinal array). Stimulation current passing from an electrode is modelled as a current source, and the resulting voltage at every node of the tissue model is computed using the admittance method. The admittance method discretises the model into voxels based on the admittance values. The admittance values for different retina layers are extracted from [28]. To reduce computational cost and maintain the accuracy of results, a non-uniform mesh with the smallest voxel size of 10 μm is utilised.

A neural network, consisting of ganglion cells, bipolar cells, AII amacrine cells, and their synapses, is constructed based on the connectomics data of rabbit retina [29]. The cells and their synapses are modelled using NEURON, a dedicated neural simulation software [30]. Mathematical models of the ionic mechanisms are used to implement the biophysical behaviour of cells and synapses. Admittance method computed voltage is interpolated at all sections of the neural model. This interpolated voltage is then applied as the extracellular stimulation in NEURON simulations. To incorporate the oscillatory behaviour of networks of cone bipolar cells and AII amacrine cells, the intrinsic properties of the neuronal model are modified to replicate the oscillatory behaviour similar to what is found in the experiments reported in [10]. Further details about the methods and modelling approach can be found in [26, 27].

Using this computational framework, we investigated how the degenerated retina responds to different stimulus waveforms. Specifically, we computed the firing patterns of the ganglion cell when stimulated using symmetric and asymmetric stimulus waveforms. Throughout this Letter, a temporal firing pattern of the ganglion cell is defined as ‘precise firing pattern’ when there is only one spike per stimulation pulse and no undesired spontaneous firing. If the cell spikes precisely once for each stimulation pulse, different types of temporal spiking patterns can be generated according to the prosthesis requirement. Therefore, we examined asymmetric biphasic waveform shapes and compared those with a symmetric biphasic waveform for suppressing spontaneous firing and generating precise ganglion cell firing patterns.

## 3 Results

### 3.1 Generating precise ganglion cell firing patterns

**Fig. 1:**
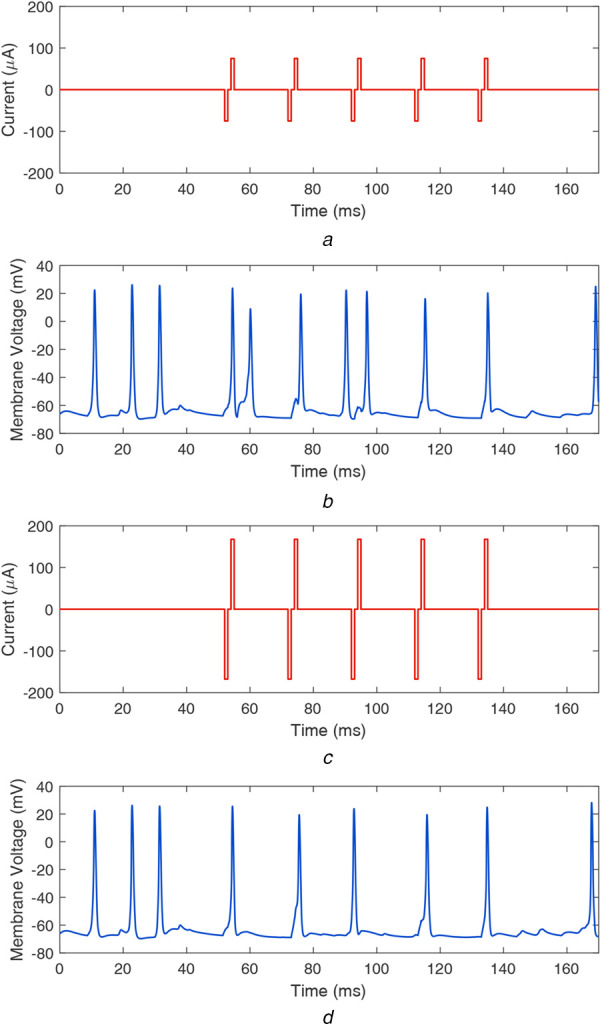
Symmetric biphasic waveforms and resulting membrane potentials at the ganglion cell *a* Waveform0 with a current magnitude of $ 75\, {\rm \mu A}$ *b* Resulting membrane potential at the ganglion cell *c* Waveform0 with a current magnitude of $ 167\, {\rm \mu A}$ *d* Resulting membrane potential at the ganglion cell

First, we analysed a symmetric biphasic current waveform, which is the most commonly used stimulus waveform in retinal implants [3, 31]. An example of such a waveform with a current magnitude of 75 μA and inter-phase gap of 1 ms is shown in Fig. 1*a*. In the rest of the Letter, this waveform is mentioned as waveform0. When stimulated by waveform0, the computed retinal response is shown in Fig. 1*b*. This plot presents the resulting temporal membrane potential at the ganglion cell, which has its soma below the stimulating electrode. It can be observed that apart from the spikes corresponding to each stimulation pulse, additional firing occurs. This additional firing of ganglion cells most likely occurs due to the spontaneous activity in the degenerated retina [32–36]. These unwanted spikes interfere with the stimulation firing patterns and could degrade the visual perception generated by retinal implants.

We investigated whether changing the current magnitude of waveform0 can subdue the spontaneous firing of ganglion cells. We increased the stimulation current from 75 μA until the additional firing of ganglion cells was suppressed. We found that for the stimulus waveform with a current magnitude of 167 μA, the additional spikes were eliminated. The stimulus waveform and corresponding firing temporal pattern are shown in Figs. 1*c* and *d*, respectively. Though this waveform could suppress the repetitive firing, it requires a higher current that might lead to tissue damage. We performed a safety analysis of the waveform by computing charge per phase and charge density per phase quantities and found that waveform0 with a current magnitude of 167 μA does not meet tissue safety criteria (further discussed in Section 3.3).

**Fig. 2:**
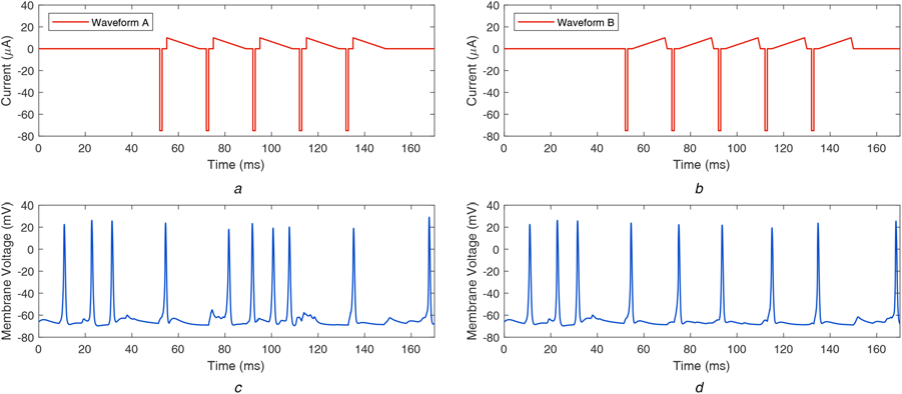
Asymmetric biphasic waveforms with decaying and rising ramp charge reversal phase, and respective retinal responses *a* Waveform A: decaying ramp charge reversal phase *b* Waveform B: rising ramp charge reversal phase *c* Resulting membrane potential at ganglion cell when stimulated by waveform A *d* Resulting membrane potential at ganglion cell when stimulated by waveform B

**Fig. 3:**
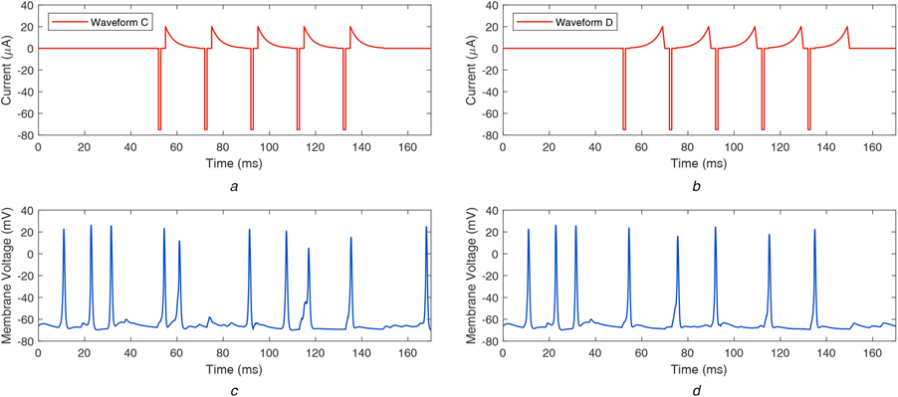
Asymmetric biphasic waveforms with decaying and rising exponential charge reversal phase, and respective retinal responses *a* Waveform C: decaying exponential charge reversal phase *b* Waveform D: rising exponential charge reversal phase *c* Resulting membrane potential at ganglion cell when stimulated by waveform C *d* Resulting membrane potential at ganglion cell when stimulated by waveform D

Next, we explored asymmetric biphasic waveform shapes that can subdue ganglion cell firing due to spontaneous activity. Choi *et al.* suggested that applying a constant current source to the bipolar cells could subdue the oscillatory behaviour of the coupled network of bipolar cells and AII amacrine cells [10]. Utilising this finding, we designed a charge-balanced asymmetric current waveform with a constant charge reversal phase and found that it can help eliminate the undesired ganglion cell firing [26]. However, it has been shown that the slower charge reversal phase can be insufficient to reverse the electrochemical processes of the stimulation phase completely; therefore, such waveforms can lead to electrode corrosion and tissue damage [37]. To counteract this effect, we designed two new stimulus waveform shapes that have a faster rate of charge reversal: a waveform with decaying ramp charge reversal phase and waveform with decaying exponential charge reversal phase. Additionally, we analysed two other waveform shapes: with rising ramp and rising exponential charge reversal phase. These waveforms are presented in Figs. 2*a* and 2*b* and Figs. 3*a* and 3*b*. In the rest of the Letter, these waveforms are called waveforms A–D as in the following manner:

- Waveform A: decaying ramp anodic phase
- Waveform B: rising ramp anodic phase
- Waveform C: decaying exponential anodic phase
- Waveform D: rising exponential anodic phase

All waveforms (A–D) first start with a cathodic phase with 75 μA current magnitude and 1 ms duration. The cathodic phase is followed by an inter-phase gap of 1 ms and the anodic phase. To prevent tissue damage and electrode corrosion, the charge transfer during anodic and cathodic phases of the stimulation pulse need to be balanced [38]. Therefore, the second phase of waveforms A–D is designed such that the charge in both phases of stimulus is equal. Waveforms A and C have a decreasing charge reversal rate, whereas waveforms B and D have an increasing charge reversal rate.

The resulting retinal responses corresponding to waveforms A–D are shown in Figs. 2*c*, 2*d* and 3*c*, 3*d*, respectively. It can be observed that waveforms A and C could not completely subdue the unwanted firing of the ganglion cell, whereas waveforms B and D successfully subdue unwanted firing and evoke one spike per stimulation pulse. Waveforms A and B work similar to waveforms C and D, respectively. It appears that the decreasing charge transfer rate has a negligible impact on suppressing undesired firing. On the other hand, the increasing charge transfer rate suppresses the spikes successfully, until another stimulation pulse is encountered.

### 3.2 Stimulation selectivity

**Fig. 4:**
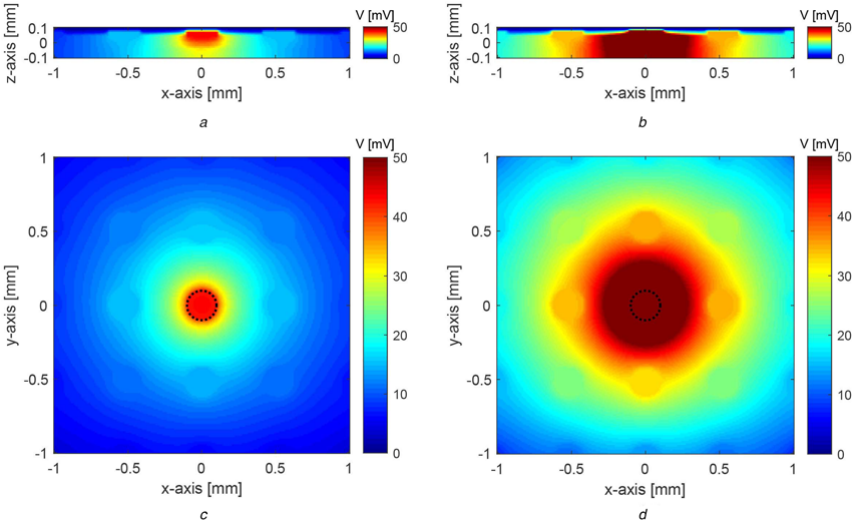
Distribution of the voltage induced by waveform B versus waveform0 with the minimum current magnitude to suppress spontaneous activity. The stimulating electrode is shown by the black dotted circle *a XZ*-slice of the voltage induced in the retina tissue due to waveform B *b XZ*-slice of the voltage induced in the retina tissue due to waveform0 *c XY*-slice of the voltage induced in the retina tissue due to waveform B *d XY*-slice of the voltage induced in the retina tissue due to waveform0

As waveforms B and D results in a similar performance, we selected waveform B for the following analyses. First, we compared the distribution of the voltage induced by waveform B against that of waveform0. The considered waveform B has a current magnitude of 75 μA, whereas, waveform0 is considered to have a current magnitude of 167 μA since this is the minimum current required to subdue the unwanted firing of cells using waveform0. The voltage induced inside the tissue (during the cathodic phase of the stimulation pulse) when stimulated by waveform B and waveform0 is plotted in Fig. 4. The electrodes are placed in the *XY*-plane, and retina layers are stacked along the *z*-axis. The *XZ*-slices shown in Figs. 4*a* and *b* pass through the centre of the stimulating electrode. The *XY*-slices of induced voltage at *z* = 0.05 mm (i.e. 0.02 mm below the electrodes) are presented in Figs. 4*c* and *d*. Owing to the differences in the current magnitude of the waveforms, required to suppress undesired firing, the induced voltage is much lower in magnitude and is more focused in the case of waveform B compared to waveform0. Consequently, when waveform B is used to provide precise firing patterns, it stimulates a smaller area compared to that of waveform0. These voltage distributions suggest that waveform B activates ganglion cells that are closer to the stimulating electrode. On the other hand, waveform0 also activates additional neighbouring ganglion cells, resulting in inferior stimulation selectivity.

### 3.3 Safety analysis

**Fig. 5:**
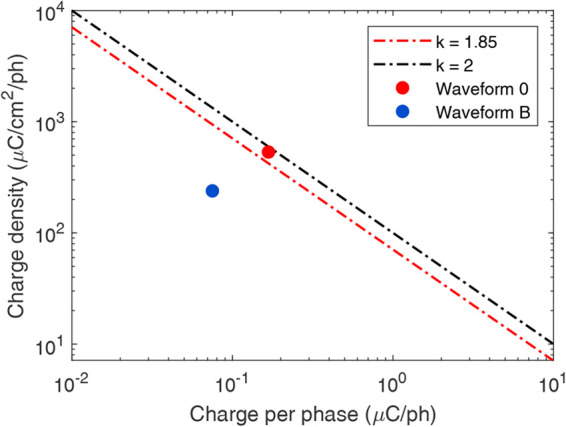
Tissue safety analysis: charge density per phase versus charge per phase plot. Red dashed line represents $k = 1.85$. Region $k \ge 1.85$ depicts damage zone and $k \lt 1.85$ depicts safe zone [39]. The red dot represents waveform0 with a current magnitude of $167\, {\rm \mu A}$, and the blue dot depicts waveform B (and waveform D) with a current magnitude of $ 75\, {\rm \mu A}$

Next, a safety analysis is performed to predict the possibility of tissue injury due to stimulation by the considered waveforms. The tissue injury induced by electrical stimulation depends on the stimulation current and electrode size. The safety evaluation can be performed mathematically by using the following equation:

(1) $$\log \lpar D\rpar = k - \log \lpar Q\rpar \eqno\lpar 1\rpar $$

where *D* is the charge density per phase [μC/cm^2^/ph], *Q* is the charge per phase [μC/ph] of the stimulus waveform, and *k* is a constant which predicts the possibility of neural injury [39]. Several experiments have suggested that the stimulus waveforms for which $k \lt 1.85$ are safe in terms of neural injury [37]. Thus, in the charge density per phase versus charge per phase plot, region $k \ge 1.85$ represents the tissue damage zone, whereas region $k \lt 1.85$ represents the safe zone. The retinal implant electrode used in our model is cylindrical, with a diameter of 200 μm and a thickness of 10 μm. The charge density versus charge per phase plot for the discussed waveforms is presented in Fig. 5. It can be observed that the symmetric biphasic waveform with a current magnitude of 167 μA lies in the tissue damage zone (depicted by a red dot in Fig. 5). However, the proposed asymmetric biphasic waveform designs (waveforms B and D) with a current magnitude of 75 μA lie in the safe zone (depicted by a blue dot in Fig. 5).

### 3.4 Effect of higher currents

**Fig. 6:**
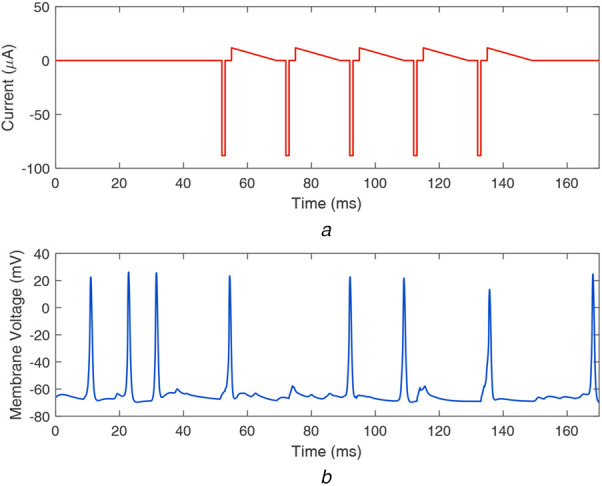
Effect of higher current magnitude of waveform A on the suppression of spontaneous firing *a* Waveform A with a current magnitude of $90\, {\rm \mu A}$ *b* Resulting membrane potential at the ganglion cell

Furthermore, we investigated if increasing the current magnitude of waveforms A and C can help in reducing the additional spikes. As shown in Fig. 6, we found that waveform A with an increased current magnitude of 90 μA helps in reducing some of the unwanted firings. Similarly, waveform C with an increased current magnitude of 113 μA helps in reducing unwanted firing to some extent (not shown here). However, these waveforms suppress some of the desired spikes (spikes due to the stimulation pulse) as well. It appears that for these waveforms, the anodic phase of the previous stimulation pulse inhibits ganglion cell firing for some time, even after the next stimulation pulse.

### 3.5 Stimulation frequency

All results in the previous sections are for the stimulation pulses separated by 20 ms (i.e. stimulation frequency of 50 Hz). We found that at stimulation frequencies <33 Hz, the stimulus waveform with a constant charge reversal phase stops suppressing all unwanted spikes. Possibly, the reason for this behaviour is that larger separation between two stimuli and the requirement of charge-balancing demands for lower stimulation current in the reversal phase. On the other hand, waveforms B and D provide more control due to additional design parameters (slope/roll-off factor). Thus, these waveforms better suppress unwanted firing at a broader frequency band.

For high-stimulation frequencies, the refractory period of neuronal spiking may not end before the arrival of the next stimulation pulse; consequently, suppression of the spikes between stimulus pulses may not be required, and traditional symmetric biphasic pulses may generate desired firing patterns. Nonetheless, retinal prosthetic systems such as the Argus II system (developed for the treatment of RP) work on a much lower stimulation frequency of 20 Hz [3, 13]. Furthermore, to closely mimic the natural visual percepts using retinal stimulation, it is essential to generate accurate spatiotemporal visual signals involving a range of stimulation frequencies. For example, the representation of a moving object will require spiking trains with different time gaps between consecutive stimulus pulses [40]. The waveforms proposed in this Letter would be useful to generate accurate firing patterns, specifically when lower stimulation frequencies are necessary and refractory periods are not sufficient to subdue undesired spikes between consecutive stimulus pulses.

### 3.6 Stimulation duration

When the stimulation duration of the considered waveforms is compared, it can be observed in Figs. 1*a*, 2*a*, 2*b* and 3*a*, 3*b* that waveform0 has a faster charge reversal rate and the charge is transferred back in 2 ms. On the other hand, waveforms A–D have slower charge reversal rates, and they take 17 ms to transfer back the charge. However, the current requirement to suppress the spontaneous activity and generate precise firing patterns is 167 μA for waveform0 and 75 μA for waveform B (and waveform D), i.e. waveform B (and waveform D) uses a 55% lesser charge than waveform0. Furthermore, due to lower current magnitude, waveform B (and waveform D) could generate precise firing patterns in smaller groups of cells and provide better stimulation selectivity, compared to waveform0.

## 4 Discussion

### 4.1 Increasing versus decreasing rate of charge reversal

The waveforms with an increasing rate of charge reversal (waveforms B and D) could suppress the spontaneous activity; whereas, waveforms with a decreasing rate of charge reversal (waveforms A and C) could not. A possible explanation behind these responses is that after the depolarisation of neurons caused by stimulus pulse, continuously increasing charge transfer during the anodic phase prolongs the time required by the neurons to recover and prepare to fire again. Thus, the action potential is not evoked due to the oscillatory behaviour of the retinal network, until the increase in charge transfer rate stops, and the next stimulus pulse arrives. In contrast, the decreasing rate of charge transfer does not exhibit such an effect.

### 4.2 Need to suppress the spontaneous firing for clinical applications

Even though currently used retinal prosthetic devices can restore useful vision to the patients of retinal degeneration diseases, the generated visual percepts are not comparable to the percepts achieved by a healthy retina. Furthermore, the performance of the prosthesis degrades with the progression of the disease. It is essential to generate precise spatiotemporal firing patterns of the ganglion cells to imitate the natural visual percepts closely. One of the primary challenges in implementing precise temporal firing patterns could be the oscillatory behaviour observed in the neural network of the degenerated retina [10–12], which leads to spontaneous firing of the ganglion cells [32–36]. This spontaneous firing of the ganglion cells could interfere with the firing patterns provided by electrical stimulation and result in degraded performance of the prosthesis [25]. To generate precise temporal firing patterns using retinal implants, it is essential to suppress the undesired firing of ganglion cells caused by the oscillatory behaviour of the degenerated retina. The waveforms presented in this Letter could generate precise temporal firing patterns by evoking just one spike per stimulation pulse and suppressing other spikes until the next stimulation pulse arrives. The electrophysiological experiments would be useful in providing further insights and validations to these simulation results. Generation of the exact shape of the stimulus waveforms using an adequately designed stimulator circuit would be crucial to investigate the scope of these waveforms for clinical applications.

## 5 Conclusions

In this work, we examined asymmetric biphasic waveforms for retinal stimulation, using a multi-scale computational framework to model the electrical stimulation of the degenerated retina. Although the proposed stimulus waveform designs have longer stimulation duration compared to the symmetric biphasic waveform, they require up to 55% lower current magnitude to suppress unwanted firing of ganglion cells and provide precise temporal spiking patterns. Furthermore, these waveforms can provide more focused stimulation and better stimulation selectivity due to their lower current requirements. Therefore, the proposed waveforms demonstrate the potential to generate precise firing patterns and improve the performance of retinal stimulation in degenerated retina.
